# SARS-CoV-2 Induces Epithelial-Enteric Neuronal Crosstalk Stimulating VIP Release

**DOI:** 10.3390/biom13020207

**Published:** 2023-01-20

**Authors:** Arun Balasubramaniam, Philip R. Tedbury, Simon M. Mwangi, Yunshan Liu, Ge Li, Didier Merlin, Adam D. Gracz, Peijian He, Stefan G. Sarafianos, Shanthi Srinivasan

**Affiliations:** 1 Division of Digestive Diseases, Department of Medicine, Emory University, Atlanta, GA 30322, USA; 2 VA Medical Center Atlanta, Decatur, GA 30033, USA; 3 Department of Pediatrics, Emory University, Atlanta, GA 30322, USA; 4 Institute for Biomedical Sciences, Center for Inflammation, Immunity and Infection, Digestive Disease Research Group, Georgia State University, Atlanta, GA 30302, USA

**Keywords:** SARS-CoV-2, diarrhea, enterocytes, enteric neurons, heat shock protein 70

## Abstract

Background: Diarrhea is present in up to 30–50% of patients with COVID-19. The mechanism of SARS-CoV-2-induced diarrhea remains unclear. We hypothesized that enterocyte–enteric neuron interactions were important in SARS-CoV-2-induced diarrhea. SARS-CoV-2 induces endoplasmic reticulum (ER) stress in enterocytes causing the release of damage associated molecular patterns (DAMPs). The DAMPs then stimulate the release of enteric neurotransmitters that disrupt gut electrolyte homeostasis. Methods: Primary mouse enteric neurons (EN) were exposed to a conditioned medium from ACE2-expressing Caco-2 colonic epithelial cells infected with SARS-CoV-2 or treated with tunicamycin (ER stress inducer). Vasoactive intestinal peptides (VIP) expression and secretion by EN were assessed by RT-PCR and ELISA, respectively. Membrane expression of NHE3 was determined by surface biotinylation. Results: SARS-CoV-2 infection led to increased expression of BiP/GRP78, a marker and key regulator for ER stress in Caco-2 cells. Infected cells secreted the DAMP protein, heat shock protein 70 (HSP70), into the culture media, as revealed by proteomic and Western analyses. The expression of VIP mRNA in EN was up-regulated after treatment with a conditioned medium of SARS-CoV-2-infected Caco-2 cells. CD91, a receptor for HSP70, is abundantly expressed in the cultured mouse EN. Tunicamycin, an inducer of ER stress, also induced the release of HSP70 and Xbp1s, mimicking SARS-CoV-2 infection. Co-treatment of Caco-2 with tunicamycin (apical) and VIP (basolateral) induced a synergistic decrease in membrane expression of Na^+^/H^+^ exchanger (NHE3), an important transporter that mediates intestinal Na^+^/fluid absorption. Conclusions: Our findings demonstrate that SARS-CoV-2 enterocyte infection leads to ER stress and the release of DAMPs that up-regulates the expression and release of VIP by EN. VIP in turn inhibits fluid absorption through the downregulation of brush-border membrane expression of NHE3 in enterocytes. These data highlight the role of epithelial-enteric neuronal crosstalk in COVID-19-related diarrhea.

## 1. Introduction

The severe acute respiratory syndrome coronavirus 2 (SARS-CoV-2), first identified in early December 2019 in Wuhan, Hubei Province, China, has spread around the world [1]. The coronavirus disease 2019 (COVID-19) has a wide range of respiratory and gastrointestinal (GI) symptom clinical symptoms, including fever, dry cough, nausea, vomiting, and diarrhea, necessitating hospitalization in intensive care units and ultimately resulting in death in severe cases [2,3,4]. It was found that up to 30–50% of MERS-CoV patients and 10.6% of SARS-CoV patients had diarrhea, which is a common symptom of coronavirus infections [4,5,6]. 

After attaching to, cleaving, and internalizing the spike protein, SARS-CoV-2 requires the angiotensin-converting enzyme 2 (ACE2) and the transmembrane protease serine 2 (TMPRSS2) to enter enterocytes (the sites of viral replication) in the luminal GI tract [7]. However, the pathophysiology of SARS-CoV-2-associated diarrhea is not yet fully understood. Usually, one of two different pathways are involved in the pathophysiology of diarrhea. The first is distinguished by cytopathic damage that frequently results in cell death, a decrease in the surface area available for digestion and absorption, and passive water flux into the intestinal lumen that is fueled by unabsorbed nutrients and causes osmotic diarrhea. The second is distinguished by a pathogenic mechanism that is functional, rather than structural, and involves enterotoxins-induced active ion secretion that affects ion transport. As a result of SARS-CoV-2 entry and replication within enterocytes, it was first hypothesized that cell death may be the pathogenic mechanism involved in SARS-CoV-2-associated diarrhea [8]. Analysis of human-derived colonic (Caco-2) cells revealed negligible cytopathic effects, despite indications of invasion and destruction by actively reproducing SARS-CoV-2 in several non-intestinal cell lines (such as Vero E6 cells) [9]. It has also been demonstrated that SARS-CoV-2 stimulates the formation of reactive oxygen species (ROS) [10]. It has been well known that endoplasmic reticulum (ER) stress is brought on by elevated levels of reactive oxygen species [11]. The ER oversees polypeptide synthesis, as well as posttranslational modification and folding of peptides to form functional proteins for cellular function or secretion. When there is an excess of misfolded proteins, it causes ER stress or induced unfolded protein responses, which has been linked to an increasing number and wide range of inherited and sporadic human diseases, ranging from gastrointestinal to neurodegenerative disorders [12]. Cells that encounter ER stress in response to pathological malfunction or cytotoxic agents can expose and release immunomodulatory DAMPs (damage-associated molecular patterns) on their surface and into the extracellular space, respectively [13]. DAMP proteins/molecules function as a signaling ligand by stimulating receptors, including toll-like receptor 2/4 (TLR2/4) [14]. This signaling can facilitate neurotransmitter modulation in enteric neurons. 

In this study, we demonstrate the novel pathophysiology of ER stress-induced diarrhea caused by SARS-CoV-2. The influence of ER stress and enteric neuron-derived vasoactive intestinal peptide (VIP) on the expression of Na^+^/H^+^ exchanger 3 (NHE3), an important transporter that mediates intestinal Na^+^/fluid absorption, was further examined.

## 2. Materials and Methods

### 2.1. Ethics and Culture of Mouse Primary Enteric Neurons 

Human colonic epithelial Caco-2 cells (ATCC#HTB-37) were cultured in DMEM (high-glucose) supplemented with 1 mM sodium pyruvate, 50 U/mL penicillin, 50 μg/mL streptomycin, and 10% fetal bovine serum. 

Myenteric neurons were isolated and cultured, as previously described [15]. In brief, enteric neurons were isolated from the intestines of 8–12 weeks old C57BL/6 mice and resuspended in Neurobasal A Medium (ThermoFisher, Waltham, MA, USA #10888022) supplemented with L-glutamine (2 mM; Invitrogen, Carlsbad, CA, USA #25030081), B27 (ThermoFisher, Waltham, MA, USA #17504044), penicillin/streptomycin (Invitrogen, #15140122), 10 ng/mL Glial cell line-derived neurotrophic factor (GDNF), and 1% FBS. Primary neurons were seeded into Matrigel-coated 6-well tissue culture plates at a density of 1 × 10^6^ cells per well. Both cell species were incubated in a humidified incubator with 5% CO_2_ at 37 °C. Primary enteric neurons at the confluence of 60–70%, were either treated with HSP70, Caco-2 cells-derived conditioned media, or co-cultured with transwell-grown Caco-2 cells. For the co-culture, fully differentiated Caco-2 cells grown on 6-well transwell filters were treated with 2 µM tunicamycin for 24 h and washed twice with PBS prior to co-culture with primary enteric neurons. All animal experiments are authorized by The Institutional Animal Care and Use Committees at Emory University and the Atlanta Veteran Affairs Medical Center. 

### 2.2. Cells, Virus Propagation, and Infection

Vero E6 (ATCC# CRL-1586), is derived from the epithelium of an African green monkey kidney and lacks type I interferon production. They were cultured in Dulbecco’s modified Eagle’s medium (DMEM, #10313-021, Gibco, Waltham, MA, USA) supplemented with 10% fetal bovine serum (FBS, #sh30396.03, lot# ag29759488, Cytiva, Marlborough, MA, USA), 2 mM L-glutamine (#25030-081, Gibco, Waltham, MA, USA), 100 units/mL penicillin, and 100 µg/mL streptomycin (#400-109, Gemini Bioproducts, West Sacramento, CA, USA). 

The mNeongreen SARS-CoV-2 reporter virus [16] was acquired from the World Reference Center for Emerging Viruses and Arboviruses (WRCEVA, Galveston, TX, USA) and then propagated in Vero-E6 cells. Virus-containing medium was harvested when a significant cytopathic effect was observed, typically 2 days post-infection. Viral stocks were tittered on Vero-E6 cells to determine infectious units per ml. Caco-2 cells, which express ACE2 and TMPRSS2, were infected at the specified multiplicities of infection (MOI) 0.001 and 0.01. 

### 2.3. Western Blotting

Primary enteric neuronal cells and intestinal Caco-2 cells, along with supernatant, were lysed in 1× Laemmli samples loading buffer (Bio-Rad, Hercules, CA, USA), supplemented with a complete protease inhibitor cocktail (Roche Diagnostics, Mannheim, Germany), and proteins separated on Criterion TGX 4-20% gels (Bio-Rad) according to recommended procedure. Separated proteins were transferred onto Immune-Blot polyvinylidene difluoride (PVDF) membranes (Bio-Rad), according to recommended procedure. The membranes were then probed with anti-BiP (rabbit, #3177, 1:1000, Cell Signaling Technology, Boston, MA, USA), anti-HSP70 (rabbit, #4872, 1:1000, Cell Signaling Technology, Boston, MA, USA), anti-CD91 (rabbit, #26387, 1:1000, Cell Signaling Technology, Boston, MA, USA), and anti-β-actin (mouse, #a5441, 1:5000, Sigma-Aldrich, St. Louis, MO, USA). Horseradish peroxidase-conjugated anti-mouse and anti-rabbit IgG (Cell Signaling Technology) secondary antibodies were used at a 1:2000 dilution. All semi-quantitative measurement of band intensity was performed using the ImageJ analysis software (US National Institutes of Health, Bethesda, MD, USA).

### 2.4. Proteomic Analysis

Caco-2 cells were infected with mNeongreen SARS-CoV-2 reporter virus 24 h. Cells were then cultured in the serum-free medium for an additional 24 h. Two hundred μL conditioned media harvested from MOCK or SARS-CoV-2 infected cells were mixed with 4 volumes of −20 °C acetone and then left at −20 °C for 1 h. The mixture was spun for 10 min at 15,000× *g*, the supernatants were decanted, and the pellets were left dry for 30 min at room temperature. Pellets were dissolved in urea buffer. Mass spectrometric analysis was then performed by the Integrated Proteomics Core Facility at Emory University following the LC-MS/MS protocol in a previous report [17]. Briefly, 35 µL protein homogenates in urea buffer were treated for 30 min with 1 mM dithiothreitol (DTT) at room temperature, followed by 5 mM iodoacetimide for 30 min in the dark. Protein samples were digested overnight with 1:50 (*w/w*) lysyl endopeptidase (Wako, Osaka, Japan). Samples were diluted with 50 mM NH_4_HCO_3_ to a final concentration of less than 2 M urea and were further digested overnight with 1:25 (*w/w*) trypsin (Promega, Madison, WI, USA). The resulting peptides were desalted with an HLB column (Waters, Milford, MA, USA) and then dried under a vacuum. Derived peptides were resuspended in a loading buffer (0.1% trifluoroacetic acid). Peptide mixtures were separated on a self-packed C18 (1.9 µm, Dr. Maisch, Ammerbuch, Germany) fused silica column (15 cm × 100 µm internal diameter (ID); New Objective, Woburn, MA, USA) attached to a Dionex 3000 RSLCnano system and were monitored on an Orbitrap fusion mass spectrometer (ThermoFisher Scientific, San Jose, CA, USA). Data were analyzed according to a published protocol [18], and they are presented as the number of peptide-spectrum match (PSM) that indicates the relative abundance of the analyzed proteins. 

### 2.5. Quantitative Real-Time Polymerase Chain Reaction (qRT-PCR)

The RNeasy Mini kit (Qiagen, Hilden, Germany) was used to collect total RNA, and the SuperScript VILO Mastermix was used to synthesize first-strand cDNA (Invitrogen, Carlsbad, CA, USA). Real-time PCR reactions were set up using TaqMan Fast Advanced Master Mix (Applied Biosystems, Foster City, CA, USA), and TaqMan primers assay for the mouse genes were provided in Table 1. Thermal cycling was performed on a StepOnePlus Real-Time PCR System (Applied Biosystems, Foster City, CA, USA). The levels of the VIP, nNOS, PRPH, and PGP9.5 mRNAs were normalized to the mRNA levels of the housekeeping gene 18s rRNA to allow comparisons among the different experimental groups using the double delta Ct method [19].

### 2.6. Enzyme-Linked Immunosorbent Assay (ELISA)

Supernatants of enteric neuronal culture were harvested, and VIP content was determined by mouse vasoactive intestinal peptide ELISA Kit (#MBS703048 MyBioSource, San Diego, CA, USA).

### 2.7. Cell Surface Biotinylation

NHE3 surface biotinylation was carried out, as previously described [20]. Briefly, fully differentiated Caco-2 cells in Transwells were treated with Tunicamycin (5 μM) on the apical side for 24 h and VIP (1 µM) on the basolateral side for 1 h. After treatment, the cells were rinsed twice in cold PBS and incubated for 10 min in borate buffer (154 mM NaCl, 7.2 mM KCl, 1.8 mM CaCl_2_, and 10 mM H_3_BO_3_, pH 9.0). Cells were then incubated for 40 min with 0.5 mg/mL NHS-SS-biotin (Pierce Biotechnology, Rockford, IL, USA) in borate buffer. Unbound biotin was quenched with Tris buffer (20 mM Tris, 120 mM NaCl, pH 7.4). Cells were then rinsed with cold PBS, scraped, and lysed in 1× cell lysis buffer (#9803, Cell Signaling Technology, Beverly, MA, USA). An aliquot of the supernatant was retained, and the total fraction representing the total cellular NHE3, and 200 µg of lysate was then incubated with streptavidin-agarose beads (Pierce Biotechnology, Rockford, IL, USA) for 2 h. Biotinylated surface proteins were then eluted by boiling the beads in 4× Laemmli buffer at 95 °C for 5 min. Densitometric analysis was performed using ImageJ software (National Institutes of Health, Bethesda, MD, USA).

### 2.8. Software and Statistical Analysis 

GraphPad Prism 9.0 (https://www.graphpad.com/ accessed on 1 November 2022; GraphPad Software, La Jolla, CA, USA) software was employed for data analysis by unpaired *t*-test, and 2-way ANOVA was appropriate. The significant difference was considered by *p*-values observation as follows: *p*-values of <0.05 (*), <0.01 (**), and <0.001 (***). The mean ± standard error of the mean (SEM) was obtained from at least three separate experiments. All graphical illustrations used in the manuscript are designed with BioRender.com.

## 3. Results

### 3.1. SARS-CoV-2 Provokes Endoplasmic Reticulum (ER) Stress in Colonic Epithelial Cells

**Figure 1:**
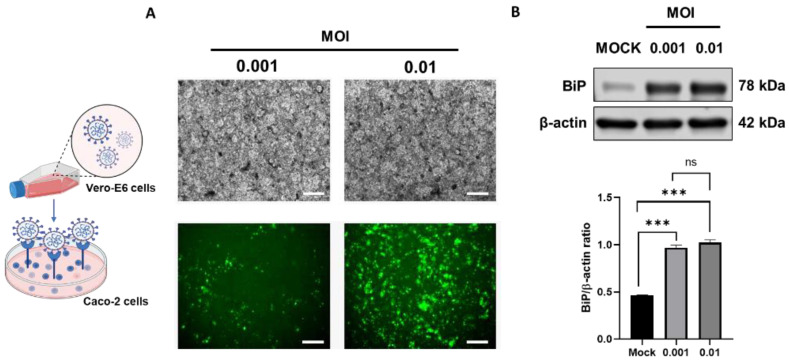
SARS-CoV-2 induces endoplasmic reticulum (ER) stress in intestinal epithelial cells. (**A**) Fluorescent imaging of Caco-2 cells that were infected with mNeongreen SARS-CoV-2 reporter virus at a multiplicity of infection (MOI) of 0.001 and 0.01. (**B**) Caco-2 cells infected at the indicated MOI were analyzed for ER stress marker BiP and loading control β-actin. The histogram shows BiP band density normalized to β-actin. Data are the mean ± SEM from three separate experiments. ns = non-significant; *** *p* < 0.001; (2-way ANOVA).

Multiple aspects of the interaction between SARS-CoV-2 and the host cell are hypothesized to contribute to the inflammatory and cytopathic pathogenesis of this viral infection. These include ER stress and the unfolded protein response (UPR) [21]. We investigated the infectious role of the SARS-CoV-2 virus in the intestinal Caco-2 cells to induce ER stress. Following virus propagation in Vero-E6 cells (Figure 1A) and infection at 0.001 and 0.01 MOI in Caco-2 cells, BiP (an HSP70 molecular chaperone localized in the endoplasmic reticulum lumen) expression (Figure 1B) was upregulated (0.001 MOI of SARS-CoV-2, 0.96 ± 0.02, *p* < 0.001 and 0.01 MOI of SARS-CoV-2, 1.02 ± 0.03, *p* < 0.001) compared to mock. Additionally, we conducted gene set enrichment analysis (GSEA) of previously published RNA-seq data from primary human enteroids infected with SARS-CoV-2 for 72 h, compared to mock-infected controls. SARS-CoV-2 infected human enteroids demonstrate significant enrichment of the Hallmark unfolded protein response gene set (Supplementary Figure S1), supporting our observation that SARS-CoV-2 is capable of triggering ER stress in Caco-2 cells.

### 3.2. SARS-CoV-2 Provokes ER Residents and DAMP Protein Secretion

**Figure 2:**
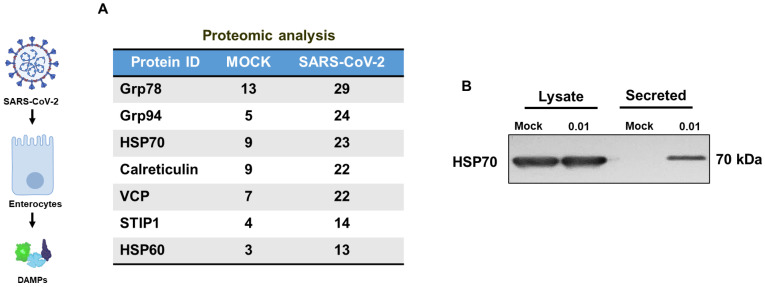
SARS-CoV-2 induces the secretion of ER-resident and DAMP proteins. (**A**) Proteomic analysis of secreted proteins from Mock and SARS-CoV-2 infected Caco-2 cells has identified multiple ER-resident proteins. The numbers denote peptide-spectrum match (PSM), indicating the relative abundance of the listed proteins. (**B**) The supernatant from Caco-2 cells treated with SARS-CoV-2 or MOCK was analyzed for HSP70 by Western blotting.

Disruptions in ER function cause ER stress, which can result in apoptosis and the release of pro-inflammatory damage-associated molecular patterns (DAMPs) molecules [22]. DAMPs participate in neurotoxic actions by acting as a signaling ligand to converse with neurons in the progression of neurodegenerative disorders, such as Alzheimer’s disease (AD) [23]. We investigated the role of SARS-CoV-2 in the induction of ER stress in enterocytes, causing the release of damage associated molecular patterns (DAMPs). To assay the secretory response of SARS-CoV-2 infected Caco2 cells, we conducted proteomic analysis (Figure 2A) of mock control vs. infected Caco-2 cells. We found that infected Caco-2 cells secreted DAMP proteins, including HSP70 and calreticulin, at much higher levels than the mock control. Western blotting analysis (Figure 2B) confirmed the upregulation of HSP70 in the conditioned media of SARS-CoV-2 treated Caco-2 cell (secreted mock, 0.553 ± 0.014; 0.01 MOI of SARS-CoV-2 secretion, 0.675 ± 0.018; *p* < 0.01) normalized to total lysate protein, indicating SARS-CoV-2 abundantly provoke ER-stress residents to release.

### 3.3. Enterocyte-Derived DAMP Proteins Induced by SARS-CoV-2 Upregulate ENS Neurotransmitters

**Figure 3:**
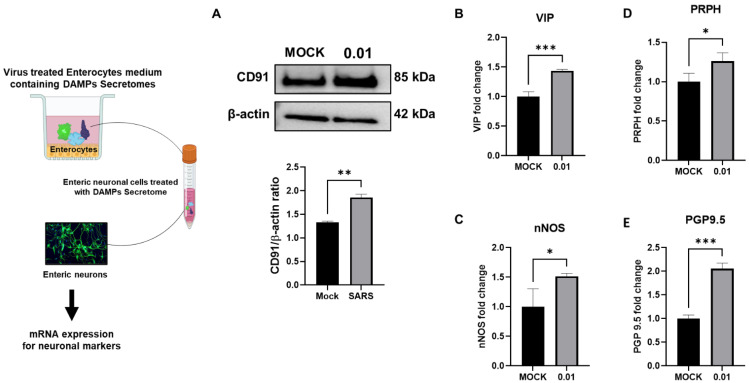
SARS-CoV-2 induced enterocyte derived DAMP proteins upregulate ENS neurotransmitters. (**A**) Western blotting analysis revealed expression of CD91 in cultured mouse enteric neurons. (**B**) The expression of VIP, (**C**) nNOS, (**D**) PRPH, and (**E**) PGP9.5 mRNA in enteric neurons. Data are the mean ± SEM from three separate experiments. * *p* < 0.05; ** *p* < 0.01; *** *p* < 0.001; (two-way ANOVA).

Under stress, DAMPs, such as high mobility group box 1 (HMGB1), have been shown to stimulate the neurotransmitter release [24]. Several neurotransmitters, including vasoactive intestinal peptide (VIP) and nitric oxide (NO) secreted by neuronal nitric oxide synthase (nNOS) neurons, have been shown to be upregulated during the progression of secretory diarrhea [25,26]. However, enteric nNOS expression has been previously reported to enhance gut motility, chronic stress, and subsequent nNOS activation, resulting in an abnormally high level of intestinal motility in the lower GI tract [27]. We used conditioned media of cultured Caco-2 cells that were infected with or not SARS-CoV-2 to treat primary enteric neuronal cell culture to test neurotransmitter mRNA expression. To confirm the entry of DAMPs in the enteric neuronal cells, we analyzed the expression of CD91, a receptor for HSP70, and calreticulin [28]. Upon treatment with supernatant containing DAMPs, enteric neuronal cells showed an increase in CD91 expression (mock, 1.36 ± 0.02; and 0.01 MOI, 1.858 ± 0.06, *p* < 0.05), (Figure 3A). The neurotransmitters VIP (0.01 MOI, 1.435 ± 0.01, *p* < 0.001), nNOS (0.01 MOI, 1.516 ± 0.02, *p* < 0.05), peripherin (PRPH) (0.01 MOI, 1.258 ± 0.06, *p* < 0.005), and PGP9.5 (0.01 MOI, 2.05 ± 0.006, *p* < 0.001) were all upregulated by the conditioned media (Figure 3B–E). These data collectively indicate that DAMP proteins are stimulated by SARS-CoV-2 and influence ENS neurotransmitters and increase the expression of CD91.

### 3.4. Enterocyte ER Stress Modulates the Release of Enteric VIP Neurotransmitters

**Figure 4:**
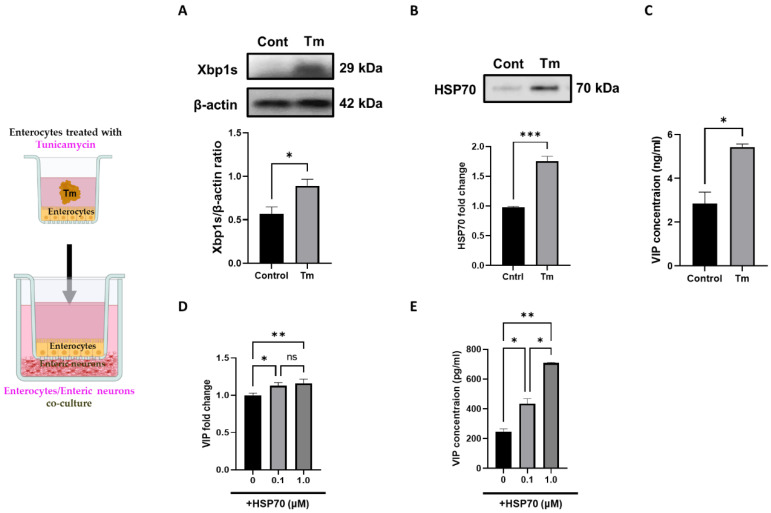
Secretomes of ER-stressed enterocytes induce VIP production in enteric neurons. (**A**) Xbp1s expression in control and Tm-treated Caco-2 cells. (**B**) HSP70 expression in the conditioned media of control and Tm-treated cells. (**C**) Mouse primary enteric neurons were co-cultured with Tm-treated Caco-2 cells for 24 h. The abundance of VIP peptide in the medium of neuronal culture was determined by ELISA. (**D**) rHSP70-treated mouse enteric neuronal cells upregulate VIP mRNA expression by RT-PCR and (**E**) VIP concentration by ELISA. Data are the mean ± SEM from three separate experiments. * *p* < 0.05; ** *p* < 0.01; *** *p* < 0.001; (two-way ANOVA).

To confirm that ER stress response of the epithelial cells upon SARS-CoV-2 infection plays a role in the enterocyte–neuronal cell communication, we tested tunicamycin (5 µM, 48 h), a pharmacological drug that inhibits N-glycosylation, to induce ER stress in Caco-2 cells, as indicated (Figure 4A,B) by the increased expression of Xbp1s (0.891 ± 0.007, *p* < 0.05) compared to control. As with findings in SARS-CoV-2 infection, HSP70 was also increased in the Tm-treated Caco-2 cells (1.754 ± 0.008, *p* < 0.001), as compared to the control samples. Next, mouse primary enteric neurons were co-cultured with Tm-treated Caco-2 cells for 24 h. The abundance of VIP peptide in the medium of neuronal culture was determined by ELISA. The data indicates the samples treated with conditioned medium treated with tunicamycin upregulate VIP expression (5.432 ± 0.135, *p* < 0.05) compared to the control (Figure 4C). To confirm the role of HSP70 in VIP induction, we treated mouse primary enteric neurons with rHSP70 at the concentrations of 0, 0.1, and 1.0 μM for 24 h and found VIP expression was increased with both 0.1 and 1.0 μM HSP70 (0.1 μM, 1.128 ± 0.04, *p* < 0.05 and 1.0 μM, 1.158 ± 0.05, *p* < 0.01) with mRNA expression by RT-PCR and VIP concentration by ELISA (0.1 μM, 434.05 ± 34.55, *p* < 0.05 and 1.0 μM, 708 ± 5.3, *p* < 0.01), indicating intestinal epithelial secretomes induced by ER-stress can upregulate VIP production.

### 3.5. VIP and ER Stress Both Cause a Synergistic Reduction in Membrane NHE3 Expression

**Figure 5:**
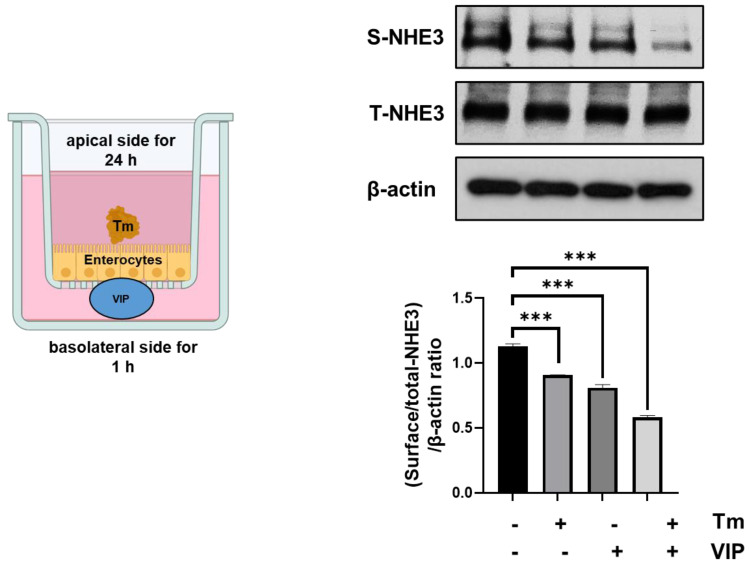
ER stress and VIP induce a synergistic decrease in membrane NHE3 expression. Tm (5 μM) was applied to the apical side of Caco-2 cells for 24 h, followed by 1 μM VIP applied to the basolateral side for 1 h. Cell surface biotinylation was used to evaluate NHE3 expression at the apical membrane and in total lysates. The histogram represents the relative ratio of the surface over total NHE3 normalized to β-actin. Data are the mean ± SEM from three separate experiments. *** *p* < 0.001; (2-way ANOVA).

A major mechanism of NHE3 regulation is through changes in subcellular localization. In the STZ-induced diabetes mouse model, intestinal fluid absorption is decreased. This drop was linked to a decrease in the expression of NHE3 and its binding proteins at the brush border membrane (BBM) [20]. The decrease in intestinal fluid absorption has been linked with the progression of diarrhea [29]. We tested whether the treatment of VIP and ER stress could contribute to the downregulation of NHE3 expression. Intestinal Caco-2 cells were treated with Tm on the apical side for 24 h, followed by VIP on the basolateral side for 1 h. NHE3 expression at the apical membrane was determined by cell surface biotinylation. Our results suggest that Tm-induced ER stress and VIP exposure synergistically decreased NHE3 expression at the cell surface (Tm, 0.906 ± 0.002, *p* < 0.001; VIP, 0.808 ± 0.02, *p* < 0.001; and Tm + VIP, 0.582 ± 0.01, *p* < 0.001) compared to vehicle, suggesting decreased NHE3 activity and Na+ absorption in Caco-2 cell model (Figure 5).

## 4. Discussion 

**Figure 6:**
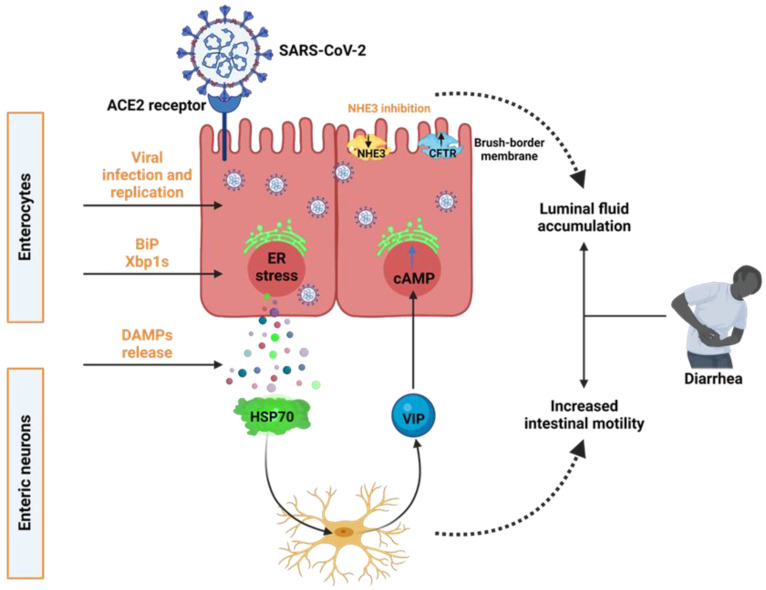
Proposed mechanism of epithelial-enteric neuronal crosstalk in SARS-CoV-2 induced diarrhea. The SARS-CoV-2 infection causes ER stress and the production of DAMPs, which increase the expression and release of VIP by enteric neurons. The presence of ER stress, together with the produced VIP, restricts fluid absorption by downregulating the brush–border membrane expression of NHE3 in enterocytes, resulting in the luminal fluid buildup and increased intestinal motility, which contributes to the onset of diarrhea.

In this study, we have shown the novel pathophysiology of SARS-CoV-2-induced ER stress-induced diarrhea. Although the consequences of SARS-CoV-2 on the respiratory system are well known, a significant number of patients develop GI problems, most often diarrhea [30]. Diarrhea has two major pathophysiological processes. The first causes osmotic diarrhea, cell injury, and a significant decrease in the digestive–absorptive surface. The second cause secretory diarrhea by activating ion secretion in response to enterotoxic effects [31,32]. VIP in the extracellular space may be associated with generating secretory diarrhea, which can be lethal with chronic persistence. Here we believe that SARS-CoV-2-induced ER stress residents can trigger ENS-stimulated VIP release to influence increased fluid secretion and reduced fluid absorption, as indicated by the decrease in membrane NHE3 expression (Figure 6).

In our present study, we found evidence that SARS-CoV-2 can induce ER stress in intestinal enterocytes and can modulate neurotransmitter release in the ENS. Further, upregulated enteric VIP neuronal modulation impacts ion transporter like NHE3 in the enterocyte brush border membrane to cause the progression of diarrhea. The gastrointestinal tract is known to have high expression of angiotensin-converting enzyme 2 (ACE2) receptors in the human body, leaving it vulnerable to direct harm from the SARS-CoV-2 cellular invasion [33]. The human body’s small intestine has the highest density of ACE2, and ACE2 is found on the luminal side of polarized enterocytes [30]. According to our findings, the mNeongreen SARS-CoV-2 reporter virus can be propagated in Vero E6 cells and be utilized to infect Caco-2 cells that are abundant in presence of ACE2 receptors. Infection and replication of SARS-CoV-2 damages tight junctions and adherent junctions in both the endothelium and the intestinal epithelium, which may result in leaky gut syndrome, local and systemic invasion of normal microbiota members, and immunological activation [34]. Viral mRNAs are translated and contribute to induced ER stress and unfolded protein responses in the rough endoplasmic reticulum in epithelial cells. The BiP/GRP78 expression increased in Caco-2 cells, a hallmark and important regulator of ER stress. It is well known that SARS-CoV-2 is prone to induce pro-inflammatory cytokine storm in affected patients. In specific terms, TNF-α is known to boost the expression of adhesion molecules on the surface of endothelial cells, platelets, and leukocytes, increasing thrombocyte adherence to arteries and starting the development of thrombi in the microcirculation of the GI and other organs. These actions enhance vascular permeability and can result in inflammation and disseminated intravascular coagulation [35]. Similarly, our results indicate that SARS-CoV-2 infection enhances the secretion of DAMPs, including HSP70 by the enterocytes. Moreover, HSP70 can capably communicate with ENS via CD91 [36], which is found to be abundantly expressed in primary myenteric cells. DAMPs can be secreted or released into enteric cells in response to a variety of stimuli, such as an infection, an inflammatory response, oxidative stress, or physical damage. While certain cytokines, such as type I interferons (IFNs), can be released in response to an infection, other DAMPs, including heat shock proteins, can also be released in response to a number of stressors, including ER stress. However, ER stress alone may not be the only cause of the rise in neurotransmitters in enteric cells.

The precise role of ENS in the pathophysiology of viral-induced secretory diarrhea is not well defined. The ENS, often known as the "brain of the gut," is critical for appropriate gastrointestinal motility. The ENS’s neuronal circuits are intended to govern gastrointestinal motility independently of central inputs [37]. The ENS neurotransmitters include nNOS, a key inhibitory neurotransmitter that mediates noradrenergic noncholinergic (NANC) transmission. nNOS generate nitric oxide (NO), which enables smooth muscle relaxation and regulates physiologic tone. Akt-phosphorylation of enteric nNOS supports normal GI motility [38]. Similarly, vasoactive intestinal peptide (VIP) is expressed in several enteric neuronal subtypes, most notably in intestinal submucosal secretomotor neurons. VIP is widely known for its ability to control physiological processes, such as intestinal secretion and motility [25]. Interestingly, in agreement with these studies, our results show the upregulation of enteric nNOS and VIP neurotransmitters, indicating intensified gut motility patterns in COVID-19 patients. This research strongly suggests that SARS-CoV-2 insults affect intestinal epithelial and enteric neuronal cross-communication, possibly being essential in neuromodulation. 

Recent research has demonstrated that ENS has its own ACE2 receptors that are impacted by viral infections, such as SARS-CoV-2 [39], and do not necessarily participate in neuromodulation via cross-communicate between enterocyte and enteric neurons. To further deepen the finding on the involvement of ER stress residents and VIP neurotransmitter, we employed a co-culture system to study cross-communication between enterocytes and enteric neuronal cells that are treated with recombinant HSP70 and well known ER stress inducer, tunicamycin, which significantly inhibits N-linked glycosylation of proteins in the ER and hence potentially produces ER stress [40]. In line with earlier findings, tunicamycin treatment elevated ER stress indicators in enterocytes, and neuronal modulation with enteric neurons demonstrated that our findings were compatible with the previous results and cross-communication between enterocytes and enteric neuronal cells. Recent studies show diarrhea is caused by decreased NHE3 activity, which may also sensitize an individual to inflammatory bowel illness [41]. Our data show that membrane NHE3 expression is decreased synergistically by VIP and ER stress in enterocyte cells.

In conclusion, our findings shed new light on SARS-CoV-2-associated diarrhea. Our data show that SARS-CoV-2 enterocyte infection causes ER stress and the release of DAMPs, both of which increase the expression and release of VIP by EN. VIP, in turn, limits fluid absorption by downregulating NHE3 brush-border membrane expression in enterocytes. These findings underscore the importance of epithelial–neuronal interaction in COVID-19-related diarrhea.

## Figures and Tables

**Table 1:** List of TaqMan primers used in this study.

Mouse Gene	Assay ID
VIP	Mm00660234_m1
nNOS	Mm01208059_m1
PRPH	Mm00449704_m1
PGP9.5	Mm00495902_m1
18s rRNA	Mm03928990_g1

## Data Availability

All data obtained or analyzed throughout this study are included in the manuscript and supplementary files.

## Supplementary Materials

[42,43,44] The following supporting information can be downloaded at: https://www.mdpi.com/article/10.3390/biom13020207/s1, Figure S1—GSEA analysis of RNA seq data from SARS-CoV-2 infected human enteroids.
