# Supplementary material for: SARS-CoV-2 Induces Epithelial-Enteric Neuronal Crosstalk Stimulating VIP Release
Source: Biomolecules. 2023 Jan 20;13(2):207. doi: 10.3390/biom13020207 (PMC9953368; doi:10.3390/biom13020207)
Supplement: Supplementary file 1 [file biomolecules-13-00207-s001.zip › biomolecules-2070400-supplementary.pdf]

Supplemental Information

SARS-CoV-2 Induces Epithelial-Enteric Neuronal Crosstalk Stimulating VIP Release

Arun Balasubramaniam [1,2], Philip R. Tedbury [3], Simon M. Mwangi [1,2], Yunshan Liu [1,2], Ge Li [1,2], Didier Merlin [2,4], Adam D. Gracz [1], Peijian He [1], Stefan G. Sarafianos [3] and Shanthi Srinivasan [1,2,*]

1 Division of Digestive Diseases, Department of Medicine, Emory University, Atlanta, GA 30322, USA; abala24@emory.edu (A.B.); smwangi@emory.edu (S.M.M.); yliu5@emory.edu (Y.L.); geli1637@gmail.com (G.L.); adam.gracz@emory.edu (A.D.G.); phe3@emory.edu (P.H.)
2 VA Medical Center Atlanta, Decatur, GA 30033, USA; dmerlin@gsu.edu
3 Department of Pediatrics, Emory University, Atlanta, GA 30322, USA; philip.tedbury@emory.edu (P.R.T.); stefanos.sarafianos@emory.edu (S.G.S.)
4 Institute for Biomedical Sciences, Center for Inflammation, Immunity and Infection, Digestive Disease Research Group, Georgia State University, Atlanta, GA 30302, USA
* Correspondence: ssrini2@emory.edu; Tel.: +1-404-727-5638; Fax: +1-404-727-5767

Supplementary method

Gene set enrichment analysis (GSEA)

A ranked gene list was generated from published differential gene expression analyses of RNA-seq data derived from primary human intestinal organoids infected with SARS-CoV-2 for 72 h vs. mock-infected controls (Supplementary Table 2, Lamers, et al 2020)[1]. GSEA was performed in GSEA 4.1.0 (https://www.gsea-msigdb.org) using the Hallmark gene set for “Unfolded Protein Response” available in the Mouse Molecular Signatures Database[2,3].

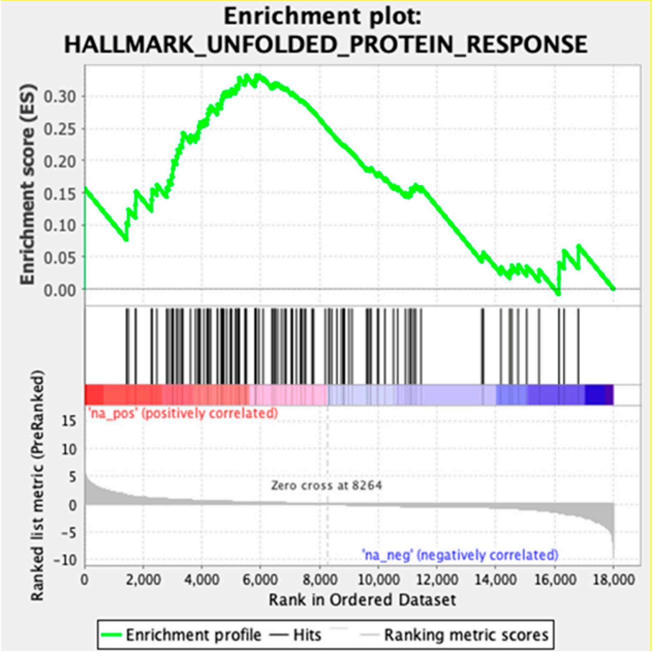

Supplementary Figure S1: GSEA analysis of RNA seq data[1] from SARS-CoV-2 infected human enteroids. RNA-seq data from primary human intestinal organoids infected for 72 h with SARS-CoV-2 against mock-infected controls.

1 Lamers, M. M. *et al.* SARS-CoV-2 productively infects human gut enterocytes. *Science* **369**, 50-54 (2020). https://doi.org:doi:10.1126/science.abc1669

2 Subramanian, A. *et al.* Gene set enrichment analysis: A knowledge-based approach for interpreting genome-wide expression profiles. *Proceedings of the National Academy of Sciences* **102**, 15545-15550 (2005). https://doi.org:doi:10.1073/pnas.0506580102

3 Mootha, V. K. *et al.* PGC-1α-responsive genes involved in oxidative phosphorylation are coordinately downregulated in human diabetes. *Nature Genetics* **34**, 267-273 (2003). https://doi.org:10.1038/ng1180
